# Influence of blue light on photoreceptors in a live retinal explant system

**Published:** 2011-04-08

**Authors:** Cora Roehlecke, Ulrike Schumann, Marius Ader, Lilla Knels, Richard H.W. Funk

**Affiliations:** 1 Institute of Anatomy, TU Dresden, Dresden, Germany; 2 CRTD/DFG-Center for Regenerative Therapies Dresden – Cluster of Excellence, TU Dresden, Dresden, Germany

## Abstract

**Purpose:**

The present study was performed to investigate the early effects of blue light irradiation of photoreceptors in retinal explant cultures.

**Methods:**

Murine retinal explant cultures were irradiated with visible blue light (405 nm) with an output power of 1 mW/cm2. Dihydroethidium was used to determine the production of reactive oxygen species. Morphological alterations of photoreceptor outer segments were determined by live imaging microscopy with mitochondrial dye JC-1. Transmission and scanning electron microscopy were used for ultrastructural evaluations. Cell death in the retina was assessed by the terminal deoxynucleotidyl transferase-mediated deoxyuridine triphosphate (dUTP) nick end labeling (TUNEL) assay method.

**Results:**

Live retinal explants displayed an increase in reactive oxygen species production, as revealed by fluorescent dihydroethidium products in photoreceptor cells after 30 min of blue light exposure. After 3 h of exposure, blue light caused disorganization of the normally neatly stacked outer segments of living photoreceptors. Ultrastructural analysis revealed breaks in the cell membrane surrounding the outer segments, especially in the middle section. The outer segments appeared tortuous, and the lamellar structures had been disrupted. TUNEL-staining revealed that long-term blue light exposure induced photoreceptor cell death.

**Conclusions:**

In vitro blue light irradiation of retinal explants is a suitable model system for investigating early ultrastructural changes, as well as damage that leads to cell death in photoreceptor cells.

## Introduction

The phototransduction cascade in retinal photoreceptors has been studied in detail [1-3]. Much less is known about the mechanisms of light-induced photoreceptor damage [4]. Acute light-induced photoreceptor cell degeneration has been studied in experimental animals for over 40 years as a model for human retinal degenerative diseases [5]. Several experiments have suggested that rhodopsin bleaching triggers both visual transduction and the pathological effects of light on photoreceptors [5-9]. In particular, short wavelength blue light is capable of damaging retinal tissue [10-13].

Several studies have investigated light-induced photoreceptor cell damage using immunohistochemical and electron microscopic analysis of the retina, typically using fixed tissue, as well as western blot analysis to quantify the levels of outer segment proteins and damage [14-22].

Previous studies have employed in vitro systems to analyze damage to photoreceptors caused by blue light [23,24]. However, photoreceptor cell compartmentalization was not assessed in these studies, for various reasons. For example, in primary dissociated cell culture, photoreceptor cells lose their morphological integrity [25,26]. The cultivation of photoreceptor cells in their physiologic environment may solve this problem. Therefore, retinal explants containing functional adult photoreceptor cells could serve as an in vitro model for the study of processes responsible for light-induced damage to the retina.

In a previous study, we established an in vitro model system to irradiate cells with blue light at a well defined wavelength and output power [27].

In the present study, we investigated the temporal order of light-induced photoreceptor damage to freshly explanted retinas. After we exposed the retinas to defined amounts of blue light, we monitored production of reactive oxygen species (ROS) and cell death in the outer nuclear layer (ONL). Moreover, we analyzed in detail photoreceptor outer segment integrity by 3D reconstruction, confocal microscopy, and electron microscopy. Freshly explanted retinas represent a model system that can be used to investigate the early events in light-induced photoreceptor cell damage.

## Methods

### Chemicals and antibodies

We obtained the following reagents: propidium iodide, 4',6-diamidino-2-phenylindole dihydrochloride (DAPI), and 2’7’-dihydro ethidium (DHE; Sigma-Aldrich, München, Germany); Dulbecco’s modified eagle medium (DMEM)/F12 and 50X B-27 supplement (Invitrogen, Darmstadt, Germany); 5,5′,6,6’-tetrachloro-1,1’,3,3′-tetraethylbenzimidazol-carbocyanine iodide (JC-1; Molecular Probes, Leiden, The Netherlands); penicillin-streptomycin and glutamine (Biochrom, Berlin, Germany); agarose (Roth, Karlsruhe, Germany); and paraformaldehyde (PFA; Merck, Darmstadt, Germany); sodium cacodylate trihydrate, osmic acid, and glutaraldehyde (SERVA Electrophoresis, Heidelberg, Germany), and EMbed 812 resin (Electron Microscopy Sciences, Hatfield, PA).

### Preparation of eyes

On postnatal day 24±4 days (shortly after weaning), C57BL/6 mice of either sex were sacrificed by cervical dislocation. Their eyes were immediately enucleated and transferred into phosphate buffered saline (PBS; components of Dulbecco’s PBS: potassium chloride, potassium dihydrogen phosphate, sodium chloride, di-sodium hydrogen). The eyeballs were punctured with a needle to create a small hole, which enabled fluid exchange, and were transferred into DMEM/F12 medium containing 10% fetal calf serum, 2% B-27 supplement, 1% penicillin-streptomycin, and 2 mM glutamine.

All experiments were conducted in accordance with the Association for Research in Vision and Ophthalmology (ARVO) statement for the use of Animals in Ophthalmic and Vision Research and in strict compliance with EU and German law (Tierschutzgesetz).

### Irradiation with blue light

The eyeballs were cultivated in almost their original form in the medium at 37 °C, with a CO_2_ level of about 5% in a cell culture incubator for different lengths of time.

Illumination was produced by an LED-based system (# LZ1–00UA05 BIN U8; LedEngin, Santa Clara, CA) that generated 405 nm blue light with an output power of 1 mW/cm2. The eyes were positioned so their corneas faced the blue light diodes. Non-irradiated eyes were used as the controls.

### Reactive oxygen species production

DHE was used to evaluate ROS production. For this purpose, eyes were irradiated with blue light for 0.5 h, 1 h, 3 h, 6 h, and 24 h at 37 °C in a cell culture incubator. After cultivation, each eyeball was cut in half through the equator to permit removal of the anterior segment and vitreous body. For ROS production imaging, the retina was gently detached from the retinal pigment epithelium and transferred to a dish (ibidi, Martinsried, Germany) for live-cell analysis in fresh medium. Next, the retinal explants were loaded with 10 µM DHE for 1 h at 37 °C in a cell culture incubator. After incubation, the samples were rinsed once in PBS and transferred to fresh medium. Live-imaging microscopy was performed at 37 °C. The images of the retinal explants were acquired using a fluorescence microscope (IX81; Olympus, Hamburg, Germany). The same detection settings were used for each set of treatments to allow direct comparison of retinal explants treated with blue light and the time-matched cultivated controls. Fluorescence images were collected using a single rapid scan. The mean fluorescence intensity was determined from the digital pictures using Cell R software from Olympus. The ratio between irradiated retinas (numerator) and non-irradiated, time-matched control retinas (denominator) was calculated to determine increases in ROS production in specific treatment groups.

### Imaging of outer segments of living photoreceptor cells

Following blue light exposure for 3 h, each eyeball was cut in half through the equator to permit removal of the anterior segment and vitreous body. Each eyecup (retina still attached to retinal pigment epithelium) was transferred to a dish for live cell analysis in fresh medium and was loaded with the conventional mitochondrial dye JC-1 (7.5 µM) for 15 min at 37 °C. The samples were then viewed using confocal laser scanning microscope imaging at 23 °C. Measurements were acquired with a TCS SP5 inverted confocal laser scanning microscope (Leica Microsystems, Wetzlar, Germany) with 405 nm, 458 nm, 477 nm, 488 nm, 514 nm, 561 nm, and 633 nm laser lines. Specimens were examined with an HCX PL APO 63X/1.2 NA water immersion objective with a working distance of 0.22 mm. Retinas stained with JC-1 were excited at 488 nm. The resulting images were acquired, stored, and visualized using Leica confocal software (Wetzlar, Germany). Image elaboration, analysis, and 3D rendering were performed using Velocity software (Perkin Elmer, Rodgau, Germany). Confocal microscopy imaging of live explants enabled differentiation between the cells present in the outer segment layer.

### Transmission electron microscopy

After blue light exposure for 3 h, 6 h, and 24 h at 37 °C in a cell culture incubator, each eyeball was cut in half through the equator to permit removal of the anterior segment and vitreous body. Next, the eyecup (retina still attached to retinal pigment epithelium) was fixed overnight at 4 °C in Karnovsky’s fixative (components: 2,5% glutaraldehyde+2,5% PFA) in 0.1 M cacodylate buffer (CB; pH 7.4).

Fixed eyecups were quartered. The tissue pieces were washed and post-fixed in 1% osmic acid prepared in 0.1 M CB (pH 7.4) for 1 h at room temperature and washed in CB. For dehydration of the samples, an ethanol series with ascending concentrations was used. The sample was infiltrated with an EMBed 812 resin overnight and then placed in an embedding mold and polymerized in an oven at 60 °C for 48 h. Ultrathin sections (65 nm) were applied to a grid.

Ultrastructural studies were conducted using an EM 906 electron microscope (Carl Zeiss, Oberkochen, Germany).

### Quantification of breaks in the surrounding membrane of outer segments

The ultrathin retina sections of at least three animals were analyzed, and around 100 photoreceptor outer segments were counted per time point and treatment. We carefully selected only rod outer segments. The counted photoreceptor outer segments were randomly distributed over the retina cross-section, but it was confirmed that the number of counted photoreceptors in the periphery was roughly the same as that of the counted photoreceptors in the middle part of the section. Every clear disruption in the surrounding membrane of the outer segment was counted as a break, was limited in size to one-third of the outer membrane, and was assigned to a respective part. Breaks were counted in three equal parts of the outer segment—in a proximal third, in a middle third, and in a distal third.

### Scanning electron microscopy

Following blue light exposure for 12 h, each eyeball was cut in half through the equator to permit removal of the anterior segment and vitreous body. The retina was gently detached from the retinal pigment epithelium and fixed overnight at 4 °C in Karnovsky’s fixative in 0.1 M CB (pH 7.4). The fixed eyecups were sectioned into strips. The tissue pieces were washed and post-fixed in 1% osmic acid prepared in 0.1 M CB (pH 7.4) for 1 h at room temperature, and washed in CB. Subsequently, they were dehydrated in ascending concentrations of acetone and were critical point-dried in liquid CO_2_. The parts of the dried retina were gold-coated (20 nm in thickness) in a sputter coater and then examined and photographed using a JSM-7500F field emission scanning electron microscope (JEOL, Eching, Germany).

### Terminal deoxynucleotidyl transferase-mediated deoxyuridine triphosphate nick end labeling assay

The terminal deoxynucleotidyl transferase-mediated deoxyuridine triphosphate (dUTP) nick end labeling (TUNEL) assay was used to detect fragmented DNA in photoreceptor cells following light exposure. After blue light exposure for 6 and 18 h, each eyeball was cut in half through the equator to permit removal of the anterior segment and vitreous body. The retina was gently detached from the retinal pigment epithelium and fixed overnight at 4 °C in 4% PFA. The fixed retina was cut into two parts and embedded in 4% agarose. Sections (30 µm) were obtained using a vibratome (VT1200 S; Leica Microsystems, Wetzlar, Germany) and then mounted and stained on glass slides. The TUNEL assay was performed according to the instruction manual of the In Situ Cell Death Detection Kit (TMR red; Roche Diagnostics, Mannheim, Germany). Subsequently, DAPI staining was conducted for 10 min. Fluorescence images were obtained using an Axio Imager Z1 microscope (Carl Zeiss). Digital pictures were acquired, stored, and visualized with Axio Vision 4.7 Software (Carl Zeiss).

Each image was acquired as a z-stack of approximately 7 µm thickness and merged for the counting of TUNEL-positive cells in the ONL. The middle of each retina was determined by using the length of the undisturbed ganglion cell layer. Sections of 100 µm were counted in central parts of the retina (ca. 200 µm from the middle) and in peripheral parts of the retina (ca. 1,000 µm from the middle) of different animals. At least eight different sections were counted per time point and treatment, and the mean value was determined.

### Statistical analysis

Values are presented as the mean±standard deviation (SD), and n represents the number of independent experiments. Unless stated otherwise in the figure legends, all assays were performed at least five times. Statistical analysis was performed by one-way ANOVA (ANOVA) with Bonferroni correction for post hoc multiple comparisons using the Statistical Package for the Social Sciences (SPSS, v. 12.0; Chicago, IL). Significance was accepted at p<0.05.

## Results

### Production of reactive oxygen species in photoreceptor cells after short-term exposure to blue light

**Figure 1:**
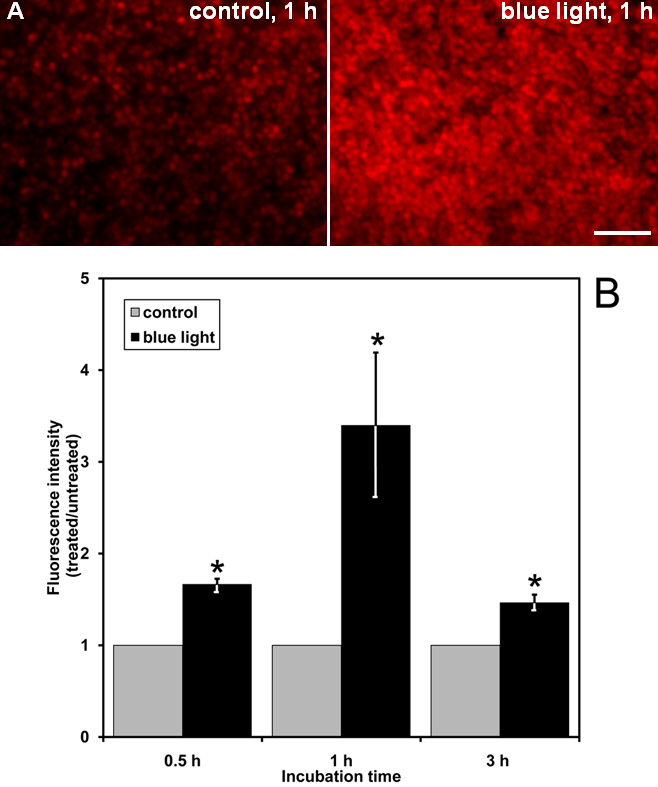
Production of intracellular reactive oxygen species is increased by blue light damage. In part **A**, representative images of reactive oxygen species production by live imaging fluorescence microscopy in photoreceptor cells of retinal explants are presented. After 1 h of blue light exposure, irradiated explants and respective non-irradiated explants (controls) were loaded with 10 µM dihydroethidium. The scale bar represents 50 µm. In part **B**, quantitative analysis of reactive oxygen species production in photoreceptor cells of retinal explants is demonstrated. Retinas were exposed to visible blue light for 0.5 h, 1 h, and 3 h. The graph displays the mean fluorescence intensity ratios of irradiated photoreceptor cell layers versus non-irradiated time-matched controls, which are normalized to 1. Bars represent the mean±standard error of mean (SEM) from n=5 independent experiments (*p<0.05).

To test for blue light-induced ROS formation in our system, we treated live retinal explants with blue light for 0.5 h, 1 h, 3 h, 6 h, and 24 h. Intracellular ROS production was measured by incubating the cells with DHE, which is commonly used to measure intracellular superoxide generation. In our system, the retinal explants displayed an increase in fluorescent products of DHE in photoreceptors following exposure to blue light. We discovered that 1 h of exposure to blue light stimulated the greatest amount of ROS formation, as evidenced by the oxidation of DHE (Figure 1). The blue light exposure induced a 1.7 fold increase in ROS production in the outer segments and ONL after 0.5 h, a 3.4 fold increase after 1 h, and a 1.5 fold increase after 3 h. After this time, the blue light exposure led to ROS production comparable to that observed in the controls (data not shown).

### Shape and structural changes in the outer segments

To image live photoreceptor cells ex vivo, we used the mitochondrial dye JC-1 according to the technique described by Bianchini et al. [28]. Briefly, the eyecup was incubated for about 20 min with JC-1 until the rods spontaneously detached from the retinal pigment epithelium. Then the retina was mounted on coverslip glass for confocal microscopy measurements with a TCS SP5 inverted confocal laser scanning microscope (Leica Microsystems). This technique resulted in staining of not only the retinal mitochondria but also the outer segments of photoreceptor cells, which are devoid of mitochondria. This additional JC-1 staining was not observed in any other cells throughout the entire living retina, as first described in Bianchini et al. [28].

**Figure 2:**
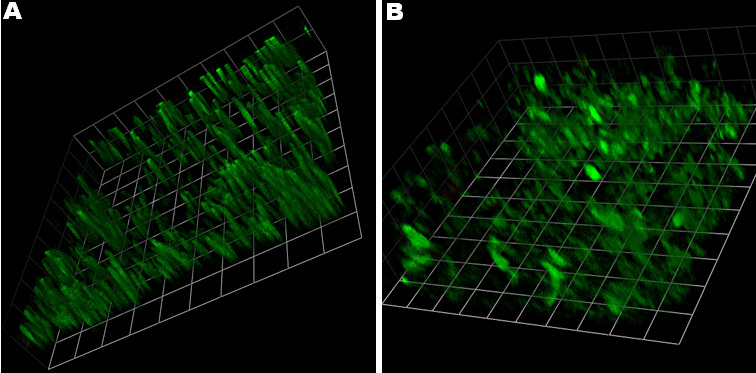
Blue light damage caused disorganization of photoreceptor outer segments. Confocal laser scanning microscope images of 5,5′,6,6’-tetrachloro-1,1’,3,3′-tetraethylbenzimidazol-carbocyanine iodide (JC-1) fluorescence in outer segments in a living murine retinal explant by 3-D reconstruction comprising serial confocal sections are presented of an undulating portion of the retina. Representative images of photoreceptor outer segments are provided for a three-hour untreated control (**A**) and a sample (**B**) that was irradiated for three hours with blue light with an output power of 1 mW/cm2. All units are 8.8 µm.

Images of living retina tissue were obtained using confocal microscopy. Figure 2 shows that the fluorescence was distributed in the outer segments of photoreceptor cells. The morphology of the outer segments was sufficiently observable to allow unambiguous identification of its structure and organization. Blue light irradiation for 3 h caused disorganization (Figure 2B) of the normally neatly stacked outer segments (OS; Figure 2A).

**Figure 3:**
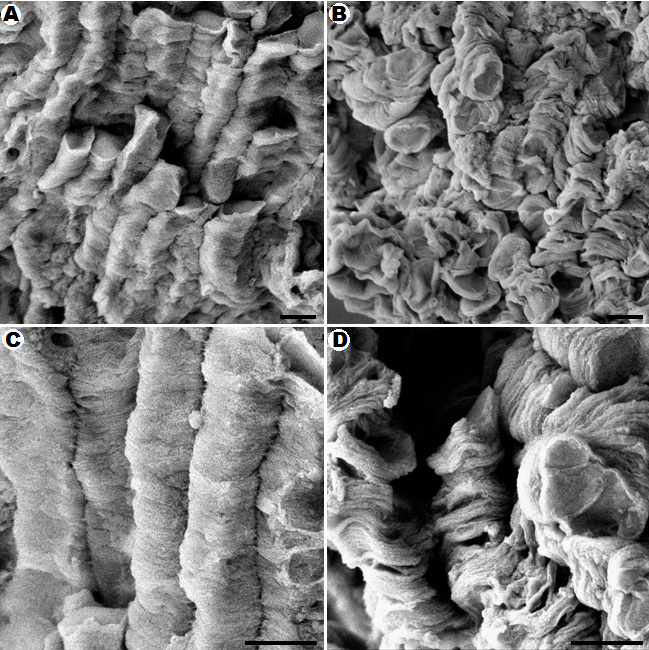
Disorganization of photoreceptor disks is caused by light damage after 12 h of blue light exposure. Scanning electron microscopy images of photoreceptor outer segments from untreated (**A**, **C**) and irradiated retinal explants (**B**, **D**) are presented. After irradiation with blue light, the outer segments of the retinas lost their shape (**B**, **D**) and were no longer as precisely stacked as the controls (**A**, **C**). The contacts between disks were lost, and the lamellar structures became disrupted (**D**). Scale bars are 1 µm.

**Figure 4:**
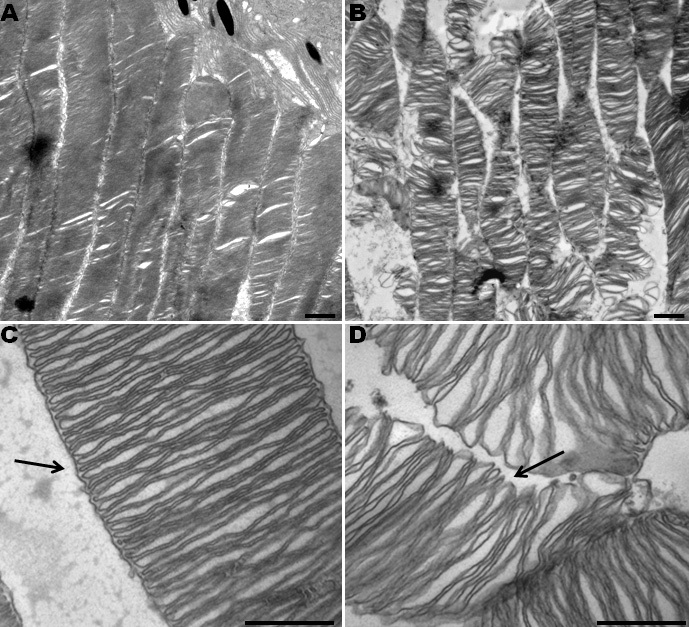
Blue light damage induced tortuous photoreceptor outer segments with disrupted lamellar structures after 24 h of blue light exposure. Transmission electron microscopy images of photoreceptors from untreated (**A**, **C**) and irradiated eyes (**B**, **D**) are presented. In part **A**, an overview of precisely stacked photoreceptor outer segments and the adjacent retinal pigment epithelium in the upper right part of the image is demonstrated. In part **B**, an overview of light-damaged photoreceptor outer segments and their disrupted lamellar structures is presented. **C**: The enveloping membrane of the outer segment continuously enclosed the disks in the control retina. **D**: The enveloping membrane was frequently interrupted or completely lost in the middle section of the outer segment, and the disks started to shift. Scale bars are 1 µm in **A** and **B**, and 500 nm in **C** and **D**.

**Figure 5:**
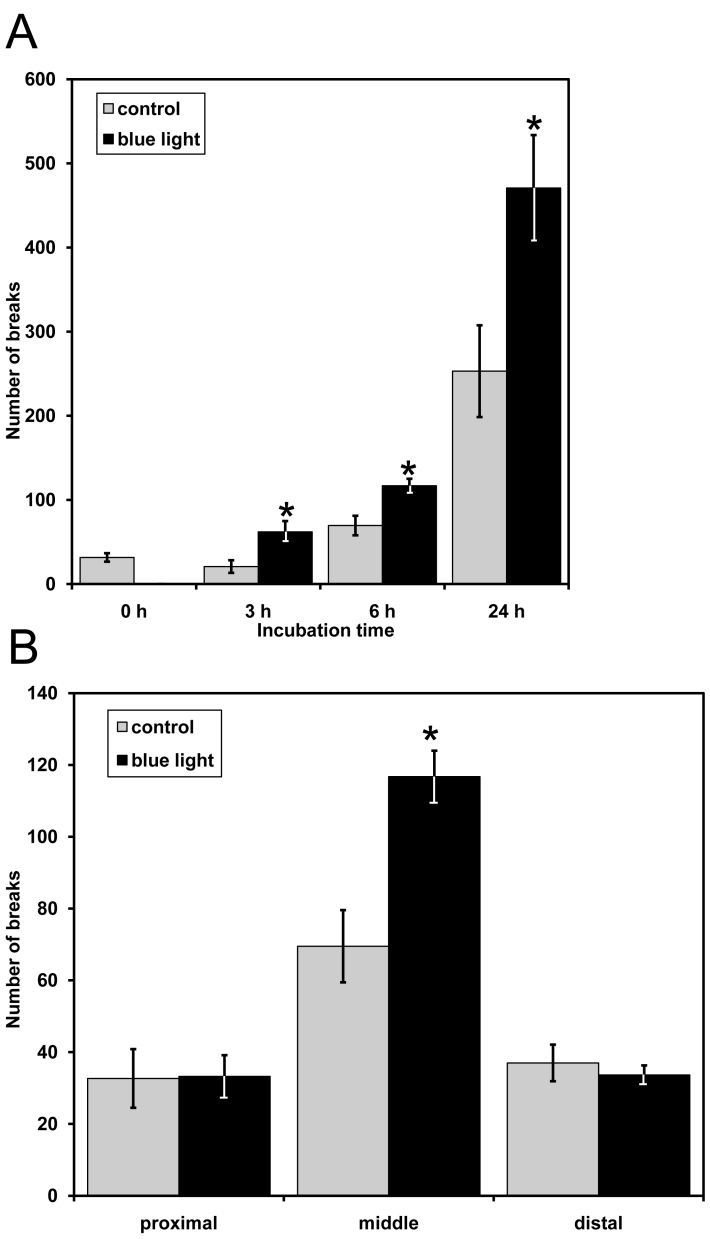
Membrane breaks in photoreceptor outer segments are caused by blue light damage. Quantitative analysis of breaks in the enveloping membrane of photoreceptor outer segments was performed. Error bars represent the mean±standard deviation (SD) from n=100 different outer segments. **A**: Eyeballs were exposed to blue light for 3 h, 6 h, and 24 h. The graph displays the number of breaks in the middle section of the outer segments. During the prolonged incubation time, the number of breaks increased in treated eyes but was consistently significantly higher than that observed in time-matched controls (*p<0.05). **B**: Eyeballs were exposed to blue light for 6 h. The graph displays the number of breaks in the proximal, middle, and distal sections of the outer segments. The number of breaks was significantly higher only in the middle section, compared to that in the controls (*p<0.05).

Scanning electron microscopy showed disorganization of the disks in the photoreceptor outer segments of irradiated retinal explants after 12 h of blue light exposure (Figure 3B,D). The outer segments lost their shape and were no longer precisely stacked, and the contacts between disks were abrogated. The outer segments appeared tortuous, and the lamellar structures became disrupted, as visualized by transmission electron microscopy (Figure 4B,D). Interestingly, this method also allowed the observation of defects in the enveloping cell membrane of outer segments (Figure 4D). An increase in cell membrane defects as the cultivation time increased was also detected in the controls, but the defects in the treated samples were significantly increased following irradiation for 3 h, 6 h, and 24 h, compared with the time-matched controls (Figure 5A). Surprisingly, most of the breaks occurred in the middle section of the outer segments after exposure to blue light (Figure 5B). Both the distal and proximal ends of the outer segments demonstrated a lower defect ratio for the surrounding cell membrane, compared to the middle section. No significant effects on cell membrane breaks in the distal and proximal ends of the outer segments were found, compared to the controls. No differences in mitochondria were detected in the inner segments (data not shown).

### Cell death in the outer nuclear layer

**Figure 6:**
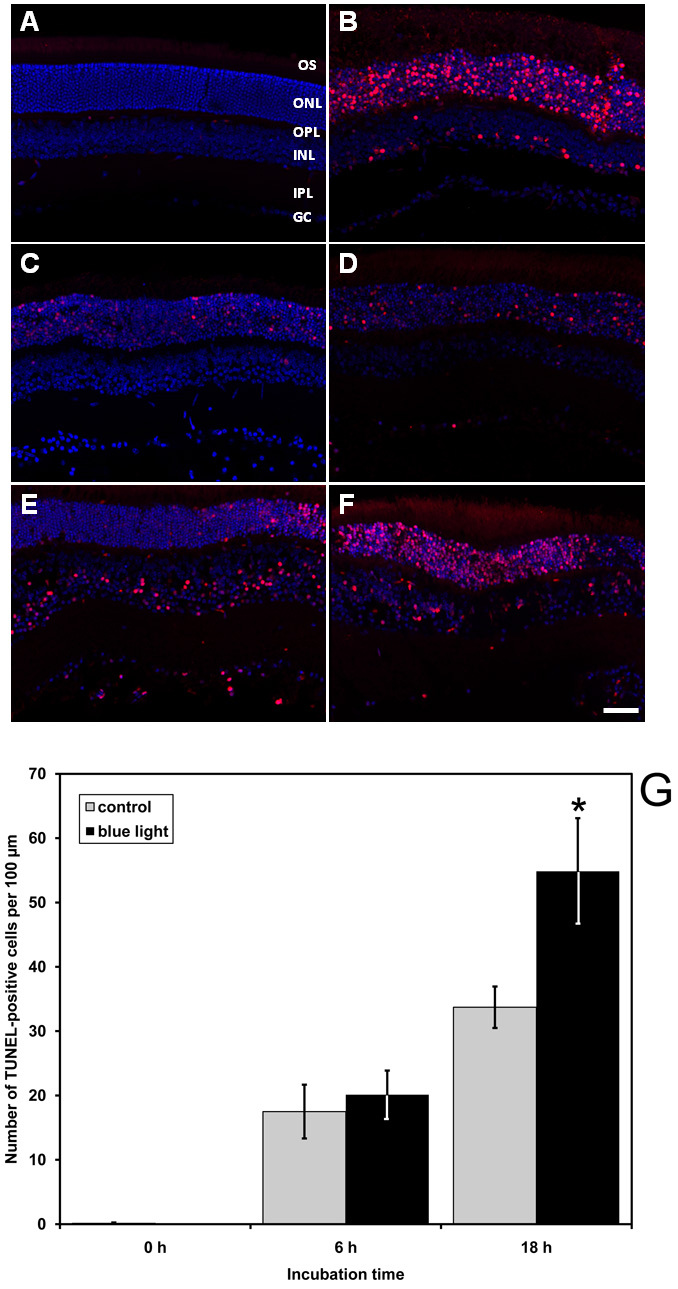
Blue light damage induced cell death in the outer nuclear layer of retinal explants. Photoreceptor cell death was quantified using the terminal deoxynucleotidyl transferase-mediated deoxyuridine triphosphate (dUTP) nick end labeling (TUNEL) assay. All vibratome sections are counterstained with 4',6-diamidino-2-phenylindole dihydrochloride (DAPI). Red represents cells with DNA strand breaks labeled with tetramethylrhodamine (TMR)-dUTP, and blue represents DAPI-stained nuclei. The figure shows retinal sections with their layers (outer segments [OS], outer nuclear layer [ONL], outer plexiform layer [OPL], inner nuclear layer [INL], inner plexiform layer [IPL], ganglion cells [GC]). **A**: The untreated retina (0 h) was incubated with complete TUNEL reaction mixture (negative control). **B**: Retinas were treated with DNase I before labeling (positive control). Retinas were not treated (**C**) or were irradiated (**D**) with visible blue light for 6 h. Cell death initiation was detected in the ONL after 6 h. Cell death initiation was detected, especially in the outer nuclear layer (ONL). Retinas were not treated (**E**) or were irradiated (**F**) with visible blue light for 18 h. The number of TUNEL-positive cells significantly increased in blue light-damaged retinas after 18 h, although some TUNEL-positive cells were detected in control explants due to the culture conditions. The scale bar represents 50 µm. In part **G**, quantitative analysis of cell death in the ONL of retinal explants after blue light exposure for 6 h and 18 h is presented. The graph displays the number of TUNEL-positive cells in the ONL of 100 µm sections from middle and peripheral parts of the retina. The mean values of at least eight different counted sections per time point and treatment are shown. TUNEL-positive cells appear initially at 6 h and increase during prolonged incubation. At 18 h, the TUNEL-positive cells in the ONL of blue light-treated eyes are significantly more numerous than the cells observed in the time-matched controls (*p<0.05). Error bars represent SD.

The TUNEL assay method was used to analyze cell death in retina layers following exposure to blue light. Cell death increased during an extended blue light treatment, especially in the ONL (Figure 6). The initiation of cell death was observed after 6 h, and this process progressed during extended blue light irradiation. Although some TUNEL-positive cells were also detected in the control samples due to the culture conditions, a significant increase in blue light-damaged retinas was observed after 18 h of irradiation. No significant changes were detected in the thickness of the ONL, compared to the control retinas.

## Discussion

In this study, we focused on the effects of blue light exposure on photoreceptors within living retinal explants of the mouse. We show for the first time that retinal explants allow direct imaging of photoreceptor cells in their physiologic environment ex vivo, following blue light exposure. Thus, this in vitro model system is suitable for direct observation of the early effects of irradiation of photoreceptor cells with visible blue light.

Recent work by Bhatt et al. [29] has shown that photoreceptor cells produce ROS in response to stress, due to the removal of trophic factors in a live retinal explant system. We used our model’s ROS detection to investigate the effect of blue light. The production of ROS was tested with DHE, which is oxidized in the presence of superoxide radicals [30].

In our system, blue light exposure caused significant ROS production in the photoreceptors. These findings are consistent with previous studies that demonstrated the ability of blue light to trigger intracellular ROS production in other retinal cells [31-33]. Only a few studies have directly investigated the relationship between blue light and ROS production in photoreceptors. Blue light exposure of isolated salamander photoreceptor ellipsoids without outer segments or distal axon terminals results in the generation of ROS [23]. Demontis et al. [24] detected an increase in light-induced oxidation in isolated rod cell fragments from frogs. Consequently, oxidative stress appears to be an early event in the process of retinal damage following light exposure. In our model system, the initial overshooting production of ROS in photoreceptor cells occurred before any noticeable structural degeneration or cell death. Increased oxidative stress is commonly interpreted as a damaging factor in the development of age related macular degeneration (AMD) [34]. Increased free radical production may also be related to excessive light exposure [13].

In our model system, blue light exposure affected the organization of photoreceptor outer segments. After only 3 h of blue light exposure, photoreceptor outer segments appeared to be disordered. This finding was confirmed by our observations of tortuous outer segments and disrupted lamellar structures using scanning electron microscopy (SEM). Therefore, the position of the outer segment tips is altered from their previous location in the retina. The modified orientation of photoreceptor cell patterns by blue light could have implications for the structural and functional integrity of photoreceptor cells. Disorganization could lead to adverse effects on the alignment of photoreceptor outer segments and could potentially disturb processes involved in visual transduction of the image projected onto the retina.

Although there is no direct published evidence linking photoreceptor cell alignment to the pathogenesis of AMD, there is considerable circumstantial and indirect evidence that suggests a possible connection. Eckmiller [35] has described a possible pathomechanism for AMD that involves a causal relationship between the cone photoreceptor alignment and a disturbed visual feedback mechanism that leads to energy depletion and apoptosis of macular photoreceptors. Via this mechanism, a disturbance in photoreceptor alignment could produce some of the alterations in central vision observed during the early stages of AMD. It is noteworthy that the initial pathological production of ROS and the altered alignment of photoreceptor cells occurred before any noticeable anatomic degeneration or cell death.

It is clear that the cones are affected in AMD, and cones have no membrane that internalizes the discs. However, similar to human rods, rod disks in mouse retinas are completely internalized and therefore physically separated from the surrounding cell membrane [36]. Interestingly, in our system, blue light exposure caused a significant disorganization of the ensheathing cell membrane of rod outer segments. What is more, following irradiation, an increase in the number of cell membrane defects was detected in the middle section of the outer segments. Thus, the surrounding cell membrane in the middle section appeared to be less stable than the surrounding cell membranes of the distal and proximal ends of the outer segments. In contrast to the ensheathing cell membrane, discontinuities in disk membranes were not observed. However, the decomposition of the surrounding cell membrane led to disorganization of the stacks connected in parallel in the outer segments, where a loss of membrane segments was common.

It is commonly suggested that light-induced damage of photoreceptor cells is initiated in the distal tips of the outer segments [4,15,37,38] and is limited by the constant renewal rate of the disks [13,39]. However, in our system, blue light exposure was capable of causing defects in surrounding cell membranes mainly in the middle part of the outer segment. That can be deduced from the mechanical properties of the very elongated outer segment: an area of weakness in its middle part. The observed damage has critical implications for the disks. It seems likely that cell membrane damage in the outer segments will have a detrimental effect on the ordered arrangement of lamellar structures of the disks and lead to misalignment of the outer segments. This finding provides an additional indication that the cell membranes of the outer segments are one of the first targets of light damage. The proposed loss of membrane segments may otherwise be due to their unique distribution of lipids. Thus, enriched outer segment plasma membrane preparations would be very useful in characterizing the components involved in blue light-induced stress. To date, no direct studies have investigated the effect of light on the surrounding cell membrane of the outer segments.

In our system, prolonged blue light exposure caused cell death in the ONL of retinal explants. These findings are consistent with the results of previous studies demonstrating that blue light can trigger intracellular ROS production and apoptosis in different retinal cell lines [31-33].

In summary, we have shown that blue light exposure can have detrimental effects on photoreceptors. An investigation of photoreceptor cells in a live retinal explant system may aid in clarifying this issue in the future. Furthermore, mouse photoreceptors are quite similar to primate photoreceptors with respect to their physical dimensions [36]. This is especially so with respect to the interphotoreceptor space, which is an appropriate model for further investigation of the sequence of pathological steps induced by blue light exposure. In further studies, we will concentrate on cones, the less occurring type of photoreceptors, in the mouse retina.

## References

[1] Lamb TD, Pugh EN (1992). A quantitative account of the activation steps involved in phototransduction in amphibian photoreceptors.. J Physiol.

[2] Nikonov S, Engheta N, Pugh EN (1998). Kinetics of recovery of the dark-adapted salamander rod photoresponse.. J Gen Physiol.

[3] Leskov IB, Klenchin VA, Handy JW, Whitlock GG, Govardovskii VI, Bownds MD, Lamb TD, Pugh EN, Arshavsky VY (2000). The gain of rod phototransduction: reconciliation of biochemical and electrophysiological measurements.. Neuron.

[4] Organisciak DT, Vaughan DK (2010). Retinal light damage: mechanisms and protection.. Prog Retin Eye Res.

[5] Noell WK, Walker VS, Kang BS, Berman S (1966). Retinal damage by light in rats.. Invest Ophthalmol.

[6] Kaitz M, Auerbach E (1979). Retinal degeneration in RCS rats raised under ambient light levels.. Vision Res.

[7] Williams TP, Howell WL (1983). Action spectrum of retinal light-damage in albino rats.. Invest Ophthalmol Vis Sci.

[8] Grimm C, Wenzel A, Hafezi F, Yu S, Redmond TM, Reme CE (2000). Protection of Rpe65-deficient mice identifies rhodopsin as a mediator of light-induced retinal degeneration.. Nat Genet.

[9] Golczak M, Kuksa V, Maeda T, Moise AR, Palczewski K (2005). Positively charged retinoids are potent and selective inhibitors of the trans-cis isomerization in the retinoid (visual) cycle.. Proc Natl Acad Sci USA.

[10] Putting BJ, Zweypfenning RC, Vrensen GF, Oosterhuis JA, van Best JA (1992). Blood-retinal barrier dysfunction at the pigment epithelium induced by blue light.. Invest Ophthalmol Vis Sci.

[11] Grimm C, Wenzel A, Williams T, Rol P, Hafezi F, Reme C (2001). Rhodopsin-mediated blue-light damage to the rat retina: effect of photoreversal of bleaching.. Invest Ophthalmol Vis Sci.

[12] Wenzel A, Grimm C, Samardzija M, Reme CE (2005). Molecular mechanisms of light-induced photoreceptor apoptosis and neuroprotection for retinal degeneration.. Prog Retin Eye Res.

[13] Wu J, Seregard S, Algvere PV (2006). Photochemical damage of the retina.. Surv Ophthalmol.

[14] Tanito M, Elliott MH, Kotake Y, Anderson RE (2005). Protein modifications by 4-hydroxynonenal and 4-hydroxyhexenal in light-exposed rat retina.. Invest Ophthalmol Vis Sci.

[15] Vaughan DK, Nemke JL, Fliesler SJ, Darrow RM, Organisciak DT (2002). Evidence for a circadian rhythm of susceptibility to retinal light damage.. Photochem Photobiol.

[16] Grignolo A, Orzalesi N, Castellazzo R, Vittone P (1969). Retinal damage by visible light in albino rats. An electron microscope study.. Ophthalmologica.

[17] O'Steen WK, Shear CR, Anderson KV (1972). Retinal damage after prolonged exposure to visible light. A light and electron microscopic study.. Am J Anat.

[18] Büchi ER, Lam TT, Suvaizdis I, Tso MO (1994). Injuries induced by diffuse photodynamic action in retina and choroid of albino rats. Morphologic study of an experimental model.. Retina.

[19] Cortina MS, Gordon WC, Lukiw WJ, Bazan NG (2005). Oxidative stress-induced retinal damage up-regulates DNA polymerase gamma and 8-oxoguanine-DNA-glycosylase in photoreceptor synaptic mitochondria.. Exp Eye Res.

[20] Lawwill T (1982). Three major pathologic processes caused by light in the primate retina: a search for mechanisms.. Trans Am Ophthalmol Soc.

[21] Shahinfar S, Edward DP, Tso MO (1991). A pathologic study of photoreceptor cell death in retinal photic injury.. Curr Eye Res.

[22] Tso MO (1987). Retinal photic injury in normal and scorbutic monkeys.. Trans Am Ophthalmol Soc.

[23] Yang JH, Basinger SF, Gross RL, Wu SM (2003). Blue light-induced generation of reactive oxygen species in photoreceptor ellipsoids requires mitochondrial electron transport.. Invest Ophthalmol Vis Sci.

[24] Demontis GC, Longoni B, Marchiafava PL (2002). Molecular steps involved in light-induced oxidative damage to retinal rods.. Invest Ophthalmol Vis Sci.

[25] Fintz AC, Audo I, Hicks D, Mohand-Said S, Leveillard T, Sahel J (2003). Partial characterization of retina-derived cone neuroprotection in two culture models of photoreceptor degeneration.. Invest Ophthalmol Vis Sci.

[26] Léveillard T, Mohand-Saïd S, Lorentz O, Hicks D, Fintz AC, Clérin E, Simonutti M, Forster V, Cavusoglu N, Chalmel F, Dollé P, Poch O, Lambrou G, Sahel JA (2004). Identification and characterization of rod-derived cone viability factor.. Nat Genet.

[27] Roehlecke C, Schaller A, Knels L, Funk RH (2009). The influence of sublethal blue light exposure on human RPE cells.. Mol Vis.

[28] Bianchini P, Calzia D, Ravera S, Candiano G, Bachi A, Morelli A, Bruschi M, Pepe IM, Diaspro A, Panfoli I (2008). Live imaging of mammalian retina: rod outer segments are stained by conventional mitochondrial dyes.. J Biomed Opt.

[29] Bhatt L, Groeger G, McDermott K, Cotter TG (2010). Rod and cone photoreceptor cells produce ROS in response to stress in a live retinal explant system.. Mol Vis.

[30] Zhao H, Kalivendi S, Zhang H, Joseph J, Nithipatikom K, Vásquez-Vivar J, Kalyanaraman B (2003). Superoxide reacts with hydroethidine but forms a fluorescent product that is distinctly different from ethidium: potential implications in intracellular fluorescence detection of superoxide.. Free Radic Biol Med.

[31] King A, Gottlieb E, Brooks DG, Murphy MP, Dunaief JL (2004). Mitochondria-derived reactive oxygen species mediate blue light-induced death of retinal pigment epithelial cells.. Photochem Photobiol.

[32] Wood JP, Lascaratos G, Bron AJ, Osborne NN (2007). The influence of visible light exposure on cultured RGC-5 cells.. Mol Vis.

[33] Osborne NN, Li GY, Ji D, Mortiboys HJ, Jackson S (2008). Light affects mitochondria to cause apoptosis to cultured cells: possible relevance to ganglion cell death in certain optic neuropathies.. J Neurochem.

[34] Bailey TA, Kanuga N, Romero IA, Greenwood J, Luthert PJ, Cheetham ME (2004). Oxidative stress affects the junctional integrity of retinal pigment epithelial cells.. Invest Ophthalmol Vis Sci.

[35] Eckmiller MS (2004). Defective cone photoreceptor cytoskeleton, alignment, feedback, and energetics can lead to energy depletion in macular degeneration.. Prog Retin Eye Res.

[36] Mustafi D, Engel AH, Palczewski K (2009). Structure of cone photoreceptors.. Prog Retin Eye Res.

[37] Bush RA, Reme CE, Malnoe A (1991). Light damage in the rat retina: the effect of dietary deprivation of N-3 fatty acids on acute structural alterations.. Exp Eye Res.

[38] Remé CE (2005). The dark side of light: rhodopsin and the silent death of vision the proctor lecture.. Invest Ophthalmol Vis Sci.

[39] Young RW (1976). Visual cells and the concept of renewal.. Invest Ophthalmol Vis Sci.

